# Household Adversity, Day to Day Experiences, and Birth/Pregnancy Complications are Associated with Delay Discounting: Findings from the Adolescent Brain Cognitive Development Study

**DOI:** 10.1007/s10802-026-01437-y

**Published:** 2026-03-02

**Authors:** I-Tzu Hung, Nathaniel S. Thomas, Brett Gelino, Justin C. Strickland, Ran Barzilay, Tyler M. Moore, Elina Visoki, Brion Maher, Julia W. Felton, Jill A. Rabinowitz

**Affiliations:** 1 Department of Psychiatry, Robert Wood Johnson Medical School, Piscataway, NJ USA; 2 Department of Psychiatry and Behavioral Sciences, Johns Hopkins University School of Medicine, Baltimore, MD USA; 3 Lifespan Brain Institute (LiBI) of the Children’s Hospital of Philadelphia, Philadelphia, PA USA; 4 Department of Psychiatry, University of Pennsylvania, Philadelphia, Philadelphia, PA USA; 5 Department of Child and Adolescent Psychiatry and Behavioral Sciences, Children’s Hospital of Philadelphia, Philadelphia, PA USA; 6 Department of Mental Health, Johns Hopkins Bloomberg School of Public Health, Baltimore, MD USA; 7 Department of Psychology, Wayne State University, Detroit, MI USA; 8 Kahlert Institute for Addiction Medicine, University of Maryland, Baltimore, MD USA

**Keywords:** Exposome, Built environment, Discounting, Adolescence, Adversity

## Abstract

Delay discounting, or the propensity to devalue rewards as the time to reward receipt increases, is a robust predictor of psychiatric and neurodevelopmental outcomes across the life course. However, less is known about environmental antecedents that may be associated with delay discounting tendencies during adolescence, a developmental period during which delay discounting behaviors are still developing. Here, we examined the relation between delay discounting and the exposome—multi-level environmental exposures experienced from conception onwards. Participants included 9,848 children (Mage = 10.94 years, SD = 0.64; 53.2% female; 72% White) from the Adolescent Brain Cognitive Development Study who completed the Adjusting Delay Discounting Task at the 1-year follow-up. Predictors included six exposome factors that captured aspects of proximal and distal environments including: positive day-to-day experiences, family values, household adversity, neighborhood poverty, birth/pregnancy complications, and state-level conservatism/rurality. Greater household adversity, lower positive day-to-day experiences, and greater birth/pregnancy complications were associated with greater delay discounting, after adjusting for age, sex, race, household income and parent education. These findings highlight potential intervention and policy targets aimed at modifying delay discounting preferences in adolescence and reducing risk for negative sequelae across development.

**Supplementary Information:**

The online version contains supplementary material available at 10.1007/s10802-026-01437-y.

## Introduction

Delay discounting, or the depreciation of the value of a reward as a function of its delay, is a decision making process that has been linked to numerous health outcomes across the developmental course (Amlung et al., 2019; DeRosa et al., 2024). Indeed, greater delay discounting (i.e., preference for smaller, immediate rewards over larger, delayed rewards) has been associated with poorer school performance (Freeney & O’Connell, 2010), and higher levels of gambling (Cosenza & Nigro, 2015), depression (Imhoff et al., 2014), body mass index (Felton et al., 2020), and substance use (Kim-Spoon et al., 2019; Stanger et al., 2012). Although there is evidence that delay discounting is a trait-like phenomenon (Odum, 2011), other work has indicated that this phenomenon is malleable, particularly during adolescence, highlighting the importance of understanding environmental factors associated with this decision making behavior during this developmental period (Rung & Madden, 2018). Such an approach may help inform intervention targets aimed at modifying discounting of delayed rewards and reducing risk for psychopathology and negative health sequelae.

Research has shown that early exposure to disadvantaged environments and experiences may promote greater delay discounting or the tendency to “get what you can, when you can.” (Andreoni & Sprenger, 2012; Del Giudice et al., 2016; Weber & Chapman, 2005). To date, most studies investigating environmental contributions to delay discounting in adolescents have focused on proximal environmental factors such as family socioeconomic status and adverse childhood experiences. For example, greater delay discounting has been associated with higher levels of household food insecurity, and lower family income and parental occupational prestige (Crandall et al., 2022; DeRosa et al., 2023; Farley & Kim-Spoon, 2017; Felton et al., 2022; Stanger et al., 2012). Similarly, greater delay discounting has also been linked to higher levels of harsh parenting, household chaos, and parental history of suicide behaviors and substance use (Liu et al., 2020; Peviani et al., 2019).

Although numerous studies have focused on the impact of proximal familial environments on delay discounting, little is known about whether distal, macro-level environmental exposures experienced across the lifespan shape decision making processes in adolescents. According to ecological systems theory, the environment is a complex system in which individuals are nested within multiple interconnected systems of influence (Bronfenbrenner, 1977). In recognition of this, there has been growing attention regarding the role of the exposome and the built environment in influencing adolescent development. Coined by Wild (2005), the exposome includes a constellation of environmental factors ranging from school and neighborhood contexts to state-level policies and chemical exposures experienced across the life course, beginning during the prenatal period onwards. The exposome has been associated with numerous adolescent cognitive, mental, and physical health outcomes, including functional brain network topography (Keller et al., 2024), neural development (Simpson-Kent et al., 2023), psychopathology (Moore et al., 2022), suicide attempt (Visoki et al., 2024), allostatic load (Hoffman et al., 2023), and self-regulation (Farley & Kim-Spoon, 2014; Roy et al., 2014; Trommsdorff, 2009). To our knowledge, only one study has examined more distal aspects of the environment in relation to decision making and delay discounting in adolescents (Felton et al., 2024). This research showed that greater perceived school and neighborhood support mitigated the effects of socioeconomic stress on delay discounting (Felton et al., 2024). Thus, research on the association between more distal environmental contexts and delay discounting is wanting.

The present study aimed to disentangle links between the exposome and delay discounting in adolescents by leveraging data drawn from the Adolescent Brain Cognitive Development Study (ABCD). We examine various environmental factors spanning prenatal, familial, school, neighborhood, and state-level policies that may influence delay discounting in adolescents (Moore et al., 2022), addressing a key limitation in prior studies that primarily relied on self-reports of the environment. Indeed, retrospective self-reports on contextual factors (e.g., childhood adversity) may be biased (e.g., (Colman et al., 2016; Hardt & Rutter, 2004), posing challenges in distinguishing whether the effects attributed to self-reported environments are due to the actual contexts themselves, or a reflection of the subjective perceptions of those environments. A better understanding of the contributions of the exposome to delay discounting has the potential to guide policy development and support targeted intervention for those at higher risk for greater delay discounting and associated negative outcomes.

## Methods

### Participants

The ABCD study is a large longitudinal study featuring 21 data collection sites across the United States focused on examining changes in brain development, child behavior, and environmental factors related to substance use engagement (Barch et al., 2018). The ABCD study employed a stratified probability sampling of schools based on participant sex, socioeconomic status, race/ethnicity, and urbanicity at each site to reduce systematic sampling biases in recruitment. The analytic sample included 9,848 children (Mage = 10.94 years, SD = 0.64; 52.6% male; 54.8% White, 13.0% Black, 19.5% Hispanic, 2.2% Asian, and 10.4% Other) who completed the delay discounting task at the 1-year follow-up and had exposome data. All participants provided informed consent at baseline. The study protocol was approved by a central IRB at the University of California, San Diego, with additional local IRB approvals at select sites.

### Phenotypic Delay Discounting Measurement

Delay Discounting was measured using an adjusting-amount delay discounting task (Du et al., 2002) assessed at the 1-year follow-up. The task includes seven randomly ordered blocks of varying delays imposed on the future reward (delays: 6 h, 1 day, 1 week, 1 month, 3 months, 1 year, and 5 years). Participants make a series of choices between two hypothetical options—one that reflects receipt of a smaller, more immediate reward (e.g., $50 now) and one that reflects the receipt of a larger, future reward (e.g., $100 after some elapsed time). Following each choice, the value of the immediately available reward is adjusted until the participant arrives at a point of indifference between the immediate and delayed rewards. In all cases, the delayed value is fixed at $100. Titrating delay discounting tasks (e.g., adjusting-amount tasks) have demonstrated moderate-to-high internal consistency, test-retest reliability, and temporal stability among adolescents and adults (Gelino et al., 2024).

In the current study, we created two metrics to index delay discounting. First, we calculated log-transformed *k*, where *k* represents the degree of discounting based on the delay (D). Indifference points form the basis of a hyperbolic curve whereby a hyperbolic discounting function g(D) = 1/(1 + *k*D) is applied to calculate *k*. Given that *k* is often skewed (Kirby & Maraković, 1996) and to be consistent with the literature on delay discounting (Gelino et al. 2025a, b), we log-transformed *k*. Second, we calculated area-under-the-curve (AUC), a value representing the area beneath the discounting curve where greater preference for the larger later reward results in greater total graphic space under a line fitted to indifference points. Although conceptually related to *k* as a depiction of discounting behavior, AUC is not theoretically tied to a particular function (e.g., hyperbolic) and thus offers a nonparametric alternative to *k*. We thereby calculated AUC to yield an additional discounting metric that is more robust to the higher rate of atypical responding, or nonsystematic responding, observed in this dataset (Gelino et al. 2025a, b). Given that AUC may disproportionately weight larger stepwise increases in assessed delays, we employed a logarithmic alternative for AUC calculation (Borges et al., 2016). Higher values of log *k* indicate greater or *steeper* delay discounting (i.e., preference for immediate rewards), whereas higher log AUC values indicate *lower* delay discounting (i.e., preference for larger, long-term rewards).

### Exposome Measurement

We leveraged exposome factors that were previously identified in factor-analytic work that used multi-level environmental data at baseline and the 1-year follow-up, including self-reports, parent-reports, and geocoded measures, to capture the complex network structure of the environment (Moore et al., 2022). Factor analyses were applied to capture both a general exposome factor and six specific factors, from which correlated factor scores were generated. We examined six specific exposome factors: household adversity (e.g., parental mental health, family poverty), neighborhood adversity (e.g., neighborhood safety, air pollution), positive day-to-day experiences (e.g., school enjoyment, acceptance by caregivers), state conservatism/ruralness (e.g., policies reflecting state-level sexism or racism), family values (e.g., parental control over substance use access, perceptions of family support), and birth/pregnancy complications (e.g., amount of prenatal care, blood oxygen complications experienced by the child at birth). Additional information regarding the specific indicators used to capture each factor can be elsewhere (Moore et al., 2022). Higher scores reflect higher levels of household adversity and neighborhood adversity, more positive day-to-day experiences, greater state conservativism/ruralism, higher family values (e.g., more monitoring and support), and greater birth/pregnancy complications.

### Statistical Analyses

Descriptive statistics and bivariate correlations were conducted to examine associations among the exposome factors and delay discounting metrics. To examine the association among each exposome factor and the delay discounting metrics (i.e., log-calculated AUC, log *k*), linear mixed effects models were conducted using the package *nlme* in R Version 4.3.1 (Pinheiro et al., 2023). We standardized all exposome factor scores (*M* = 0, *SD* = 1) such that coefficients reflect the change in the raw scale of the delay discounting outcome associated with a 1-*SD* change in the corresponding exposome measure. All models included a random effect for family identifier, nested within a random effect for ABCD data collection site, to account for participant relatedness and site-level clustering.

Several linear mixed effects models were conducted. In the first set of models, each exposome domain was tested individually in relation to delay discounting metrics. Models adjusted for child age at the 1-year follow-up, sex, parent-reported child race, household income, and parent education to account for developmental, socioeconomic, and socio-structural factors. We selected variables we hypothesized would strongly influence delay discounting and that may confound the relationship between the exposome factors and the outcomes of interest (Button et al., 2023; Kahn et al., under review; Owen et al., 2022). We standardized the exposome factor scores to provide an interpretable scale for them. Standardization was not applied to the delay discounting variables. Thus, our coefficient estimates are partially standardized, reflecting raw change in Log AUC/Log K corresponding to a 1 standard deviation change in the corresponding exposome factor. A false discovery rate (FDR) threshold of 0.05 was applied to account for multiple test comparisons. Marginal *R*^*2*^ was also reported to reflect the incremental variance each predictor accounted for in the outcome. Given our interest in examining the relative contributions of the exposome to delay discounting, we conducted a second set of models that included the six exposome domains in a single model as predictors of the delay discounting outcomes; these models were adjusted for the same covariates as the first set of models. We tested for multi-collinearity by calculating the variance inflation factor (VIF) (Fox & Monette, 1992) for each predictor. If a VIF > 5 was observed for any of the exposome factors, we excluded those predictors from these models.

We also conducted sensitivity analyses to account for participants who showed unusual or inconsistent patterns in how they valued delayed rewards (i.e., non-systematic responding). Non-systematic responding has been uniquely associated with some demographic subgroups and thus may meaningfully characterize individual differences in delay discounting (Gelino et al. 2025a, b; Johnson and Bickel 2008). Thus, we conduced sensitivity analyses including number of non-systematic reversals as a covariate. This variable is defined as the numbers of times an individual rated a reward at a longer delay as more valuable than the same reward at a shorter delay, reflecting atypical patterns.

## Results

Descriptive statistics of the analytic sample are reported in Table 1. The sample was socioeconomically diverse, with 25% of parents reporting a household annual income of ≤$49,999 and 40.5% reporting a household annual income ≥$100,000. Similarly, 15% of parents reported a high school education or less, whereas 26.5% reported an advanced degree.

**Table 1:** Descriptive statistics of the analytic sample

Variables	M (SD) or *N* (%)
Age in years	10.94 (0.64)
Sex (male)	5181 (52.6%)
Race/Ethnicity	
White	5400 (54.8%)
Black	1280 (13%)
Hispanic	1920 (19.5%)
Asian	220 (2.2%)
Other	1027 (10.4%)
Missing	1 (0.01%)
Household Income	
<$5,000	275 (2.8%)
$5,000 - $11,999	305 (3.1%)
$12,000 - $15,999	204 (2.1%)
$16,000 - $24,999	392 (4%)
$25,000 - $34,999	519 (5.3%)
$35,000 - $49,999	761 (7.7%)
$50,000 - $74,999	1253 (12.7%)
$75,000 - $99,999	1382 (14%)
$100,000 - $199,999	2886 (29.3%)
>=$200,000	1105 (11.2%)
Missing	766 (7.8%)
Educational Attainment	
High School or Less	1482 (15%)
Some College	2834 (28.8%)
College	2912 (29.6%)
Advanced Degree	2608 (26.5%)
Missing	12 (0.1%)
Log-calculated AUC	0.71 (0.2)
Log *k*	−5.49 (3.64)
Number of Non-Systematic Reversals	0.65 (0.8)

Correlations among exposome domains and delay discounting metrics are displayed in Table 2. Several significant positive (*r* range from 0.06 to 0.37) and negative (*r* range from − 0.39 to − 0.06) correlations were observed between exposome factor scores. Household adversity, neighborhood adversity, state conservatism/ruralness, and family values were negatively correlated with log-calculated AUC (*r* range from − 0.03 to − 0.15). Positive day-to-day experiences were positively correlated with log-calculated AUC (*r* =.11). Mirroring these results, log *k* was positively correlated with household adversity (*r* =.09) and neighborhood adversity (*r* =.11), but negatively correlated with positive day-to-day experiences (*r* = −.09).

**Table 2:** Correlations [95% confidence Intervals] of exposome factors and delay discounting metrics

Variable	1	2	3	4	5	6	7	8	9
1. Household Adversity	--								
2. Neighborhood Adversity	0.37* [0.35, 0.38]	--							
3. Positive Day-to-Day Experiences	−0.39* [−0.40, −0.37]	−0.19* [−0.21, −0.17]	--						
4. State Conservatism/Ruralness	0.23* [0.21, 0.25]	0.12* [0.10, 0.14]	−0.12* [−0.14, −0.10]	--					
5. Family Value	−0.06* [−0.08, −0.04]	0.06* [0.04, 0.08]	0.00 [−0.02, 0.02]	0.18* [0.16, 0.20]	--				
6. Birth/Pregnancy Complications	0.02 [0.00, 0.04]	−0.26* [−0.28, −0.24]	0.01 [−0.01, 0.03]	0.02 [0.00, 0.03]	0.02 [0.00, 0.04]	--			
7. Number of Non-Systematic Reversals	0.14* [0.12, 0.16]	0.16* [0.14, 0.18]	−0.09* [−0.11, −0.07]	0.07* [0.05, 0.09]	0.05* [0.03, 0.07]	0.00 [−0.02, 0.02]	--		
8. Log-calculated AUC	−0.13* [−0.15, −0.11]	−0.15* [−0.17, −0.13]	0.11* [0.10, 0.13]	−0.04* [−0.06, −0.02]	−0.03* [−0.05, −0.01]	0.00 [−0.02, 0.02]	−0.38* [−0.40, −0.36]	--	
9. Log K	0.09* [0.07, 0.11]	0.11* [0.09, 0.13]	−0.09* [−0.11, −0.07]	0.02 [0.00, 0.04]	0.01 [−0.01, 0.03]	0.00 [−0.02, 0.02]	0.26* [0.24, 0.28]	−0.92* [−0.93, −0.92]	--

95% confidence intervals are presented in brackets. Statistically significant correlations are marked with *

Table 3 shows the results from the linear mixed effect models with exposome domains examined in separate models in relation to the delay discounting outcomes. After adjusting for child age, sex, parent-reported child race, household income and parental education (Model 1), greater household adversity (log *k β* = 0.184, *p*FDR = 1.61E-04, *ΔR²* = 0.186%; log-calculated AUC *β* = −0.012, *p*FDR = 9.56E-06, *ΔR²* = 0.250%), lower positive day-to-day experiences (log *k β* = −0.231, *p*FDR = 4.31E-08, *ΔR²* = 0.378%; log-calculated AUC *β* = 0.014, *p*FDR = 1.90E-09, *ΔR²* = 0.461%), and greater birth/pregnancy complications (log-calculated AUC *β* = −0.006, *p*FDR = 0.012, *ΔR²* = 0.090%) were associated with steeper delay discounting (i.e., preference for immediate, smaller rewards).

**Table 3:** Associations of exposome factors with delay discounting metrics

	Log k					Log-Calculated AUC				
	β	se	*p*	FDR- adjusted *p*	Marginal *R*^2^(%)	β	se	*p*	FDR- adjusted *p*	Marginal *R*^2^(%)
*Model 1*										
Household Adversity	**0.184**	**0.046**	**5.36E-05**	**1.61E-04**	**0.186**	**−0.012**	**0.003**	**2.39E-06**	**9.56E-06**	**0.250**
Neighborhood Adversity	0.064	0.053	0.231	0.309	0.034	−0.005	0.003	0.122	0.182	0.065
Positive Day-to-Day Experiences	**−0.231**	**0.040**	**7.18E-09**	**4.31E-08**	**0.378**	**0.014**	**0.002**	**1.58E-10**	**1.90E-09**	**0.461**
State Conservatism/Ruralness	0.004	0.039	0.941	0.941	−0.004	−0.002	0.003	0.454	0.496	0.022
Family Values	−0.072	0.039	0.067	0.115	0.043	0.002	0.002	0.282	0.338	0.015
Birth/Pregnancy Complications	0.088	0.040	0.029	0.058	0.061	**−0.006**	**0.002**	**0.005**	**0.012**	**0.090**
*Model 2*										
Household Adversity	**0.092**	**0.049**	**0.018**	--	**0.034**	**−0.006**	**0.003**	**0.018**	--	**0.049**
Neighborhood Adversity	0.050	0.055	0.227	--	0.020	−0.004	0.003	0.227	--	0.037
Positive Day-to-Day Experiences	**−0.201**	**0.042**	**1.62E-07**	--	**0.258**	**0.012**	**0.002**	**1.62E-07**	--	**0.305**
State Conservatism/Ruralness	−0.012	0.051	0.826	--	−0.007	−0.001	0.003	0.826	--	−0.001
Family Values	−0.049	0.040	0.665	--	0.021	0.001	0.002	0.665	--	0.003
Birth/Pregnancy Complications	**0.090**	**0.041**	**0.006**	--	**0.068**	**−0.006**	**0.002**	**0.006**	--	**0.087**

Bold values indicate statistical significance. Model 1 adjusted for child age, sex, parent-reported child race, household income, and parent education and included exposome factors in separate models. Model 2 adjusted for the same covariates and included all the exposome factors in the same model. FDR-adjusted *p* values are reported for Model 1

Model 2 included all the exposome factors in the same model as predictors of log *k* and log-calculated AUC. Prior to running the models, we examined the presence of multicollinearity among the exposome factors and covariates. We observed a VIF < 5 suggesting low levels of multi-collinearity (maximum VIF = 2.54 for parent education; among exposome factors, maximum VIF = 1.86 for neighborhood adversity); thus, all exposome variables were included in models. After controlling for child age, sex, parent-reported child race, household income, and parent education, greater household adversity (log *k β* = 0.092, *p* =.018, *ΔR²* = 0.034%; log-calculated AUC *β* = −0.006, *p* =.018, *ΔR²* = 0.049%), lower positive day-to-day experiences (log *k β* = −0.201, *p* = 1.62E-07, *ΔR²* = 0.258%; log-calculated AUC *β* = 0.012, *p* = 1.62E-07, *ΔR²* = 0.305%), and greater birth/pregnancy complications (log *k β* = 0.090, *p* =.006, *ΔR²* = 0.068%; log-calculated AUC *β* = −0.006, *p* =.006, *ΔR²* = 0.087%) were significantly associated with higher delay discounting.

Sensitivity analyses that further adjusted for number of non-systematic reversals revealed results similar to the main analyses (Supplemental Table 1). In models that included each individual exposome factor and adjusted for age, sex, parent-reported child race, household income, parent education, and number of non-systematic reversals, the associations of delay discounting with household adversity and positive day-to-day experiences remained significant. In addition, family values became significantly associated with log *k* (*β* = −0.091, *p*FDR = 0.040, *ΔR²* = 0.070%), whereas the associations of birth/pregnancy complications with log *k* (*p*FDR = 0.151) and log-calculated AUC (*p*FDR = 0.068) were no longer significant. The model that including all exposome factors revealed that lower positive day-to-day experiences were significantly associated with greater delay discounting (log *k β* = −0.178, *p* = 1.18E-05, *ΔR²* = 0.202%; log-calculated AUC *β* = 0.010, *p* = 2.53E-06, *ΔR²* = 0.214%). Birth/pregnancy complications were significantly associated with greater delay discounting reflected in log-calculated AUC (*β* = −0.004, *p* =.038, *ΔR²* = 0.061%) only.

## Discussion

The current study examined the associations between exposome factors (i.e., multi-level environmental exposures, including state conservatism/ruralness) and delay discounting in adolescents using data from the ABCD Study. Our results suggest that adolescents who were exposed to higher levels of household adversity, had fewer positive daily experiences, and experienced greater birth/pregnancy complications displayed a stronger preference for smaller, immediate rewards. The finding of household adversity being a stronger predictor of youth decision making than neighborhood-level disadvantage is consistent with a previous study examining the relation between exposome factors and psychopathology (Pries et al., 2022). The stronger association of delay discounting with household adversity compared to neighborhood adversity is also supported by a recent systematic review, which found that more proximal environments (e.g., parenting, trauma exposure) evidenced more robust associations with youth delay discounting than economic or neighborhood factors (Felton et al., under review). Additionally, a negative association between positive day-to-day experiences and delay discounting is consistent with a recent paper demonstrating a negative bivariate relation between delay discounting and perceived neighborhood support (Felton et al., 2024). It may be that positive childhood experiences, in contrast to environmental instability, promote a sense of stability and social support that allow youth to focus on attainment of future rewards; however, further research is needed to replicate and expand on these relations.

This study is the first to demonstrate an association between birth/pregnancy complications (e.g., premature birth) and elevated youth delay discounting, although these results are supported by literature examining relations between delay discounting and closely related constructs. Specifically, several recent reviews have highlighted associations between the number of pregnancy complications and prenatal risk factors with increased risk for the development of attention deficit/hyperactivity disorder (ADHD) in offspring (Bitsko et al., 2024; Serati et al., 2017). Youth with ADHD, in turn, evidence significantly steeper rates of delay discounting (Jackson & MacKillop, 2016). Additionally, mothers with ADHD are more likely to experience pregnancy complications (Walsh et al., 2022), representing one plausible pathway for the noted intergenerational transmission of specific decision making repertoires (Peviani et al., 2019).

In sensitivity analyses that included each individual exposome predictor that further adjusted for the number of non-systematic reversals, we observed an attenuation in links between household adversity and delay discounting. Although little research has been conducted on the construct of non-systematic responding, existing studies suggest that it is a distinct, yet related, construct to delay discounting (Gelino et al. 2025a, b). Evidence indicates that families in which parents or children exhibit non-systematic responding tend to have lower educational attainment and income and receive social welfare support (Button et al., 2023). Thus, non-systematic responding may be a proxy indicator of broader contextual factors associated with more disadvantaged environments, which may explain why the associations of delay discounting with more negative environments (i.e., household adversity and birth/pregnancy complications) were diminished. Family values were significantly associated with log k after controlling for non-systematic responding (*β* = −0.091); however, this association attenuated and was no longer significant when all exposome domains were included, indicating its marginal relation with delay discounting. Future research is needed to better understand the underlying mechanisms linking non-systematic responding in delay discounting tasks to exposome factors.

There are some limitations of the study worth acknowledging. While the current study provides critical information on the cross-sectional relation between exposome factors and delay discounting in early adolescence, longitudinal research is needed to evaluate these associations across development. For instance, the unexpected correlations observed between higher family values (e.g., stricter rules against substance use) and lower delay discounting may reflect parental practices adapting to children’s regulatory tendencies and inhibitory control, constructs that are associated with delay discounting. Future longitudinal approaches that dynamically model these constructs over time will allow for causal conclusions regarding the relationship between the exposome and delay discounting. In addition, rates of delay discounting peak before plateauing in middle adolescence (Felton et al., 2020; Steinberg et al., 2009); therefore, it is important to examine how exposome factors impact developmental changes in delay discounting through late adolescence and emerging adulthood. Future studies will benefit from more waves of data collected and released by the ABCD project. Lastly, although delay discounting is heritable (Thorpe et al., 2024), the currently study did not account for genetic influences that might moderate environmental effects. Future studies should examine potential gene-environment interplay on delay discounting.

### Clinical Implications

Findings from this research suggest one pathway by which adverse childhood experiences (parental mental illness, family poverty) and birth complications negatively impact decision making which, in turn, are associated with maladaptive health outcomes. These results also add to the growing understanding of the pernicious role of early childhood adversity and the potential for large-scale public health efforts to identify at-risk youth and effectively target prevention efforts (Dube, 2018). These results also support a growing consensus on the importance of increasing positive experiences across early development to improve healthy decision-making and positive health outcomes (Bethell et al., 2019). Consistent with behavioral economics models of health promotion (Bickel et al., 2016), improved decision-making may be one pathway by which increasing the availability of positive experiences in the environment leads to healthier outcomes (e.g., Murphy et al., 2025). Thus, promoting positive school climates, improving family relations, and mitigating prenatal health issues, all represent plausible treatment targets for reducing impulsive decision making and its associated risks (Del Giudice et al., 2016; Kim-Spoon et al., 2019; Scholten et al., 2019). Future studies could incorporate additional individual-level factors that have been empirically or theoretically been linked to delay discounting, such as psychopathology (e.g., ADHD), and neurological history (e.g., head injury) that may influence the relationship between exposome factors and delay discounting tendencies. Such an approach may refine risk profiles associated with greater delay discounting, thus informing future clinical prevention and intervention efforts.

## Conclusions

This study advances our understanding of adolescent decision making by demonstrating the association of the exposome with delay discounting. Leveraging a large-scale, epidemiologically informed study with comprehensive measures of environmental factors, our findings reveal that proximal and distal environmental factors may collectively shape decision making patterns. Future research is needed to determine whether addressing these environmental characteristics and experiences promotes lower discounting of rewards and, in turn, more positive health outcomes across development.

## Supplementary Information

Below is the link to the electronic supplementary material.

Supplementary file 1 (DOCX 31.9 KB)

## References

[CR1] Amlung, M., Marsden, E., Holshausen, K., Morris, V., Patel, H., Vedelago, L., Naish, K. R., Reed, D. D., & McCabe, R. E. (2019). Delay discounting as a transdiagnostic process in psychiatric disorders: A meta-analysis. *JAMA Psychiatry,**76*(11), 1176–1186. 10.1001/jamapsychiatry.2019.2102 31461131 10.1001/jamapsychiatry.2019.2102 PMC6714026

[CR2] Andreoni, J., & Sprenger, C. (2012). Risk preferences are not time preferences. *American Economic Review*, *102*(7), 3357–3376.

[CR3] Barch, D. M., Albaugh, M. D., Avenevoli, S., Chang, L., Clark, D. B., Glantz, M. D., Hudziak, J. J., Jernigan, T. L., Tapert, S. F., Yurgelun-Todd, D., Alia-Klein, N., Potter, A. S., Paulus, M. P., Prouty, D., Zucker, R. A., & Sher, K. J. (2018). Demographic, physical and mental health assessments in the adolescent brain and cognitive development study: Rationale and description. *Developmental Cognitive Neuroscience,**32*, 55–66. 10.1016/j.dcn.2017.10.010 29113758 10.1016/j.dcn.2017.10.010 PMC5934320

[CR4] Bethell, C., Jones, J., Gombojav, N., Linkenbach, J., & Sege, R. (2019). Positive childhood experiences and adult mental and relational health in a statewide sample: Associations across adverse childhood experiences levels. *JAMA Pediatrics,**173*(11), Article e193007. 10.1001/jamapediatrics.2019.3007 31498386 10.1001/jamapediatrics.2019.3007 PMC6735495

[CR5] Bickel, W. K., Moody, L., & Higgins, S. T. (2016). Some current dimensions of the behavioral economics of health-related behavior change. *Preventive Medicine,**92*, 16–23. 10.1016/j.ypmed.2016.06.002 27283095 10.1016/j.ypmed.2016.06.002 PMC5085840

[CR6] Bitsko, R. H., Holbrook, J. R., O’Masta, B., Maher, B., Cerles, A., Saadeh, K., Mahmooth, Z., MacMillan, L. M., Rush, M., & Kaminski, J. W. (2024). A systematic review and meta-analysis of prenatal, birth, and postnatal factors associated with attention-deficit/hyperactivity disorder in children. *Prevention Science,**25*(Suppl 2), 203–224. 10.1007/s11121-022-01359-3 35303250 10.1007/s11121-022-01359-3 PMC9482663

[CR7] Borges, A. M., Kuang, J., Milhorn, H., & Yi, R. (2016). An alternative approach to calculating Area-Under-the-Curve (AUC) in delay discounting research. *Journal of the Experimental Analysis of Behavior,**106*(2), 145–155. 10.1002/jeab.219 27566660 10.1002/jeab.219

[CR8] Bronfenbrenner, U. (1977). Toward an experimental ecology of human development. *American Psychologist*, *32*(7), 513–531. 10.1037/0003-066X.32.7.513

[CR9] Button, A. M., Paluch, R. A., Schechtman, K. B., Wilfley, D. E., Geller, N., Quattrin, T., Cook, S. R., Eneli, I. U., & Epstein, L. H. (2023). Parents, but not their children, demonstrate greater delay discounting with resource scarcity. *BMC Public Health,**23*(1), Article 1983. 10.1186/s12889-023-16832-z 37828503 10.1186/s12889-023-16832-z PMC10568819

[CR10] Colman, I., Kingsbury, M., Garad, Y., Zeng, Y., Naicker, K., Patten, S., Jones, P. B., Wild, T. C., & Thompson, A. H. (2016). Consistency in adult reporting of adverse childhood experiences. *Psychological Medicine,**46*(3), 543–549. 10.1017/s0033291715002032 26511669 10.1017/S0033291715002032

[CR11] Cosenza, M., & Nigro, G. (2015). Wagering the future: Cognitive distortions, impulsivity, delay discounting, and time perspective in adolescent gambling. *Journal of Adolescence,**45*, 56–66. 10.1016/j.adolescence.2015.08.015 26363842 10.1016/j.adolescence.2015.08.015

[CR12] Crandall, A. K., Madhudi, N., Osborne, B., Carter, A., Williams, A. K., & Temple, J. L. (2022). The effect of food insecurity and stress on delay discounting across families: A COVID-19 natural experiment. *BMC Public Health,**22*(1), Article 1576. 10.1186/s12889-022-13969-1 35986265 10.1186/s12889-022-13969-1 PMC9388997

[CR13] Del Giudice, M., Gangestad, S. W., & Kaplan, H. S. (2016). Life history theory and evolutionary psychology. *The handbook of evolutionary psychology: Foundations, Vol. 1* (2nd ed, pp. 88–114). John Wiley & Sons, Inc.

[CR14] DeRosa, J., Rosch, K. S., Mostofsky, S. H., & Nikolaidis, A. (2023). Developmental deviation in delay discounting as a transdiagnostic indicator of risk for child psychopathology. *Journal of Child Psychology and Psychiatry*. 10.1111/jcpp.13870 37524685 10.1111/jcpp.13870 PMC10828118

[CR15] DeRosa, J., Rosch, K. S., Mostofsky, S. H., & Nikolaidis, A. (2024). Developmental deviation in delay discounting as a transdiagnostic indicator of risk for child psychopathology. *Journal of Child Psychology and Psychiatry,**65*(2), 148–164. 10.1111/jcpp.13870 37524685 10.1111/jcpp.13870 PMC10828118

[CR16] Du, W., Green, L., & Myerson, J. (2002). Cross-cultural comparisons of discounting delayed and probabilistic rewards. *The Psychological Record*, *52*(4), 479–492.

[CR17] Dube, S. R. (2018). Continuing conversations about adverse childhood experiences (ACEs) screening: A public health perspective. *Child Abuse & Neglect,**85*, 180–184. 10.1016/j.chiabu.2018.03.007 29555095 10.1016/j.chiabu.2018.03.007

[CR19] Farley, J. P., & Kim-Spoon, J. (2017). Parenting and adolescent self-regulation mediate between family socioeconomic status and adolescent adjustment. *Journal of Early Adolescence,**37*(4), 502–524. 10.1177/0272431615611253 28348448 10.1177/0272431615611253 PMC5365151

[CR18] Farley, J. P., & Kim-Spoon, J. (2014). The development of adolescent self-regulation: Reviewing the role of parent, peer, friend, and romantic relationships. *Journal of Adolescence,**37*(4), 433–440. 10.1016/j.adolescence.2014.03.009 24793391 10.1016/j.adolescence.2014.03.009 PMC4021015

[CR22] Felton, J. W., Rabinowitz, J. A., Sadler, R. C., Hampton, T., Sosnowski, D. W., Lejuez, C. W., & Yi, R. (2024). Environmental support moderates the association of socioeconomic distress and delay discounting across adolescence. *Journal of Youth and Adolescence,**53*(12), 2695–2705. 10.1007/s10964-024-02051-1 39023841 10.1007/s10964-024-02051-1 PMC13254626

[CR21] Felton, J. W., Kahn, G., Johnson, J., Ali, H., Saleh, S., Maher, S., Strickland, J., Cheong, J., Yi, R., & Rabinowitz, J. A. (Under review). A Systematic Review and Meta-Analysis of the Impact of Environmental Disadvantage on Youth Delay Discounting. 10.1017/S0954579425100886 PMC12825953 41221819

[CR20] Felton, J. W., Collado, A., Ingram, K., Lejuez, C. W., & Yi, R. (2020). Changes in delay discounting, substance use, and weight status across adolescence. *Health Psychology,**39*(5), 413–420. 10.1037/hea0000833 31916829 10.1037/hea0000833 PMC7169948

[CR23] Felton, J. W., Rabinowitz, J. A., Strickland, J. C., Maher, B. S., Summers, M., Key, K., Johnson, J. E., & Yi, R. (2022). Social vulnerability, COVID-19 impact, and decision making among adults in a low-resource community. *Behavioural Processes,**200*, Article 104668. 10.1016/j.beproc.2022.104668 35667640 10.1016/j.beproc.2022.104668 PMC9164510

[CR24] Fox, J., & Monette, G. (1992). Generalized Collinearity Diagnostics. *Journal of the American Statistical Association,**87*(417), 178–183. 10.1080/01621459.1992.10475190

[CR25] Freeney, Y., & O’Connell, M. (2010). Wait for it: Delay-discounting and academic performance among an Irish adolescent sample. *Learning and Individual Differences,**20*(3), 231–236. 10.1016/j.lindif.2009.12.009

[CR27] Gelino, B. W., Schlitzer, R. D., Reed, D. D., & Strickland, J. C. (2024). A systematic review and meta-analysis of test-retest reliability and stability of delay and probability discounting. *Journal of the Experimental Analysis of Behavior,**121*(3), 358–372. 10.1002/jeab.910 38499476 10.1002/jeab.910 PMC11078611

[CR26] Gelino, B. W., Rabinowitz, J. A., Maher, B. S., Felton, J. W., Yi, R., Novak, M. D., Sanchez-Roige, S., Palmer, A. A., & Strickland, J. C. (2025a). Delay discounting data in the Adolescent Brain Cognitive Development (ABCD) study: Modeling and analysis considerations. *Experimental and Clinical Psychopharmacology*. 10.1037/pha0000766 39992757 10.1037/pha0000766 PMC12097935

[CR28] Gelino, B. W., Stone, B. M., Kahn, G. D., Strickland, J. C., Felton, J. W., Maher, B. S., Yi, R., & Rabinowitz, J. A. (2025). From error to insight: Removing non-systematic responding data in the delay discounting task may introduce systematic bias. *Journal of Experimental Child Psychology,**256*, Article 106239. 10.1016/j.jecp.2025.106239 40186956 10.1016/j.jecp.2025.106239

[CR29] Hardt, J., & Rutter, M. (2004). Validity of adult retrospective reports of adverse childhood experiences: Review of the evidence. *Journal of Child Psychology and Psychiatry,**45*(2), 260–273. 10.1111/j.1469-7610.2004.00218.x 14982240 10.1111/j.1469-7610.2004.00218.x

[CR30] Hoffman, K. W., Tran, K. T., Moore, T. M., Gataviņš, M. M., Visoki, E., DiDomenico, G. E., Schultz, L. M., Almasy, L., Hayes, M. R., Daskalakis, N. P., & Barzilay, R. (2023). Allostatic load in early adolescence: Gene / environment contributions and relevance for mental health. *medRxiv*. 10.1101/2023.10.27.2329767438076956

[CR31] Imhoff, S., Harris, M., Weiser, J., & Reynolds, B. (2014). Delay discounting by depressed and non-depressed adolescent smokers and non-smokers. *Drug and Alcohol Dependence,**135*, 152–155. 10.1016/j.drugalcdep.2013.11.014 24360649 10.1016/j.drugalcdep.2013.11.014

[CR32] Jackson, J. N., & MacKillop, J. (2016). Attention-deficit/hyperactivity disorder and monetary delay discounting: A meta-analysis of case-control studies. *Biological Psychiatry: Cognitive Neuroscience and Neuroimaging,**1*(4), 316–325. 10.1016/j.bpsc.2016.01.007 27722208 10.1016/j.bpsc.2016.01.007 PMC5049699

[CR33] Johnson, M. W., & Bickel, W. K. (2008). An algorithm for identifying nonsystematic delay-discounting data. *Experimental and Clinical Psychopharmacology,**16*(3), 264–274. 10.1037/1064-1297.16.3.264 18540786 10.1037/1064-1297.16.3.264 PMC2765051

[CR34] Kahn, G., Bounoua, N., Strickland, J. C., Hung, I. T., Maher, B. S., Rabinowitz, J. A., & Felton, J. W. Identifying significant environmental predictors of change in delay discounting among adolescents in the ABCD study. (Under review). 10.1037/pha0000851 PMC13488959 42133374

[CR35] Keller, A. S., Moore, T. M., Luo, A., Visoki, E., Gataviņš, M. M., Shetty, A., Cui, Z., Fan, Y., Feczko, E., Houghton, A., Li, H., Mackey, A. P., Miranda-Dominguez, O., Pines, A., Shinohara, R. T., Sun, K. Y., Fair, D. A., Satterthwaite, T. D., & Barzilay, R. (2024). A general exposome factor explains individual differences in functional brain network topography and cognition in youth. *Developmental Cognitive Neuroscience,**66*, Article 101370. 10.1016/j.dcn.2024.101370 38583301 10.1016/j.dcn.2024.101370 PMC11004064

[CR36] Kim-Spoon, J., Lauharatanahirun, N., Peviani, K., Brieant, A., Deater‐Deckard, K., Bickel, W. K., & King‐Casas, B. (2019). Longitudinal pathways linking family risk, neural risk processing, delay discounting, and adolescent substance use. *Journal of Child Psychology and Psychiatry,**60*(6), 655–664. 30809804 10.1111/jcpp.13015 PMC6791121

[CR37] Kirby, K. N., & Maraković, N. N. (1996). Delay-discounting probabilistic rewards: Rates decrease as amounts increase. *Psychonomic Bulletin & Review,**3*(1), 100–104. 10.3758/bf03210748 10.3758/BF03210748 24214810

[CR38] Liu, S., Cui, Z., Duprey, E., Kogan, S., & Oshri, A. (2020). Adverse parenting is indirectly linked to delayed reward discounting via blunted RSA reactivity: The protective role of a shift-and-persist coping strategy. *Adversity and Resilience Science*. 10.1007/s42844-020-00012-8

[CR39] Moore, T. M., Visoki, E., Argabright, S. T., Didomenico, G. E., Sotelo, I., Wortzel, J. D., Naeem, A., Gur, R. C., Gur, R. E., Warrier, V., Guloksuz, S., & Barzilay, R. (2022). Modeling environment through a general exposome factor in two independent adolescent cohorts. *Exposome*. 10.1093/exposome/osac010 10.1093/exposome/osac010 PMC9798749 36606125

[CR40] Murphy, J. G., Acuff, S. F., Buck, A. C., Campbell, K. W., & MacKillop, J. (2025). Reward deprivation is associated with elevated alcohol demand in emerging adults. *Journal of the Experimental Analysis of Behavior,**123*(1), 30–40. 10.1002/jeab.4229 39542832 10.1002/jeab.4229 PMC13469893

[CR41] Odum, A. L. (2011). Delay discounting: Trait variable? *Behavioural Processes,**87*(1), 1–9. 10.1016/j.beproc.2011.02.007 21385637 10.1016/j.beproc.2011.02.007 PMC3266171

[CR42] Owens, M. M., Hahn, S., Allgaier, N., MacKillop, J., Albaugh, M., Yuan, D., Juliano, A., Potter, A., & Garavan, H. (2022). One-year predictions of delayed reward discounting in the adolescent brain cognitive development study. *Experimental and Clinical Psychopharmacology,**30*(6), 928–946. 10.1037/pha0000532 34914494 10.1037/pha0000532

[CR43] Peviani, K. M., Kahn, R. E., Maciejewski, D., Bickel, W. K., Deater-Deckard, K., King-Casas, B., & Kim-Spoon, J. (2019). Intergenerational transmission of delay discounting: The mediating role of household chaos. *Journal of Adolescence,**72*, 83–90. 10.1016/j.adolescence.2019.03.002 30875564 10.1016/j.adolescence.2019.03.002 PMC6450567

[CR44] Pinheiro, J., Bates, D., & R Core Team. (2023). &. *nlme: Linear and Nonlinear Mixed Effects Models.* In (Version 3.1–162) https://CRAN.R-project.org/package=nlme

[CR45] Pries, L.-K., Moore, T. M., Visoki, E., Sotelo, I., Barzilay, R., & Guloksuz, S. (2022). Estimating the association between exposome and psychosis as well as general psychopathology: Results from the ABCD study. *Biological Psychiatry Global Open Science,**2*(3), 283–291. 10.1016/j.bpsgos.2022.05.005 36325038 10.1016/j.bpsgos.2022.05.005 PMC9616253

[CR46] Roy, A. L., McCoy, D. C., & Raver, C. C. (2014). Instability versus quality: Residential mobility, neighborhood poverty, and children’s self-regulation. *Developmental Psychology,**50*(7), 1891–1896. 10.1037/a0036984 24842459 10.1037/a0036984 PMC4727396

[CR47] Rung, J. M., & Madden, G. J. (2018). Experimental reductions of delay discounting and impulsive choice: A systematic review and meta-analysis. *Journal of Experimental Psychology: General,**147*(9), 1349–1381. 10.1037/xge0000462 30148386 10.1037/xge0000462 PMC6112163

[CR48] Scholten, H., Scheres, A., De Water, E., Graf, U., Granic, I., & Luijten, M. (2019). Behavioral trainings and manipulations to reduce delay discounting: A systematic review. *Psychonomic Bulletin & Review,**26*, 1803–1849. 31270766 10.3758/s13423-019-01629-2 PMC6863952

[CR49] Serati, M., Barkin, J. L., Orsenigo, G., Altamura, A. C., & Buoli, M. (2017). Research review: The role of obstetric and neonatal complications in childhood attention deficit and hyperactivity disorder - a systematic review. *Journal of Child Psychology and Psychiatry,**58*(12), 1290–1300. 10.1111/jcpp.12779 28714195 10.1111/jcpp.12779

[CR50] Simpson-Kent, I. L., Gataviņš, M. M., Tooley, U. A., Boroshok, A. L., McDermott, C. L., Park, A. T., Delgado Reyes, L., Bathelt, J., Tisdall, M. D., & Mackey, A. P. (2023). Multilayer network associations between the exposome and childhood brain development. *bioRxiv*. 10.1101/2023.10.23.563611 37961103 10.1101/2023.10.23.563611 PMC10634748

[CR51] Stanger, C., Ryan, S. R., Fu, H., Landes, R. D., Jones, B. A., Bickel, W. K., & Budney, A. J. (2012). Delay discounting predicts adolescent substance abuse treatment outcome. *Experimental and Clinical Psychopharmacology,**20*(3), 205–212. 10.1037/a0026543 22182419 10.1037/a0026543 PMC3906638

[CR52] Steinberg, L., Graham, S., O’Brien, L., Woolard, J., Cauffman, E., & Banich, M. (2009). Age differences in future orientation and delay discounting. *Child Development,**80*(1), 28–44. 10.1111/j.1467-8624.2008.01244.x 19236391 10.1111/j.1467-8624.2008.01244.x

[CR53] Thorpe, H. H. A., Cupertino, R. B., Pakala, S. R., Fontanillas, P., Jennings, M. V., Yang, J., Meredith, J. J., Greenwood, T., Bianchi, S. B., Vilar-Ribó, L., Niarchou, M., andMe Research, T., Elson, S. L., Ideker, T., Davis, L. K., MacKillop, J., deWit, H., Gustavson, D. E., Mallard, T. T., Palmer, A. A., & Sanchez-Roige, S. (2024). Genome-wide association study of delay discounting in 134,935 individuals identifies novel loci and transdiagnostic associations across mental and physical health. *MedRxiv*. 10.1101/2024.09.27.24314244. 2024.2009.2027.24314244.

[CR54] Trommsdorff, G. (2009). Culture and development of self-regulation. *Social and Personality Psychology Compass*, *3*(5), 687–701. 10.1111/j.1751-9004.2009.00209.x. https://doi.org/https://

[CR55] Visoki, E., Moore, T. M., Zhang, X., Tran, K. T., Ly, C., Gataviņš, M. M., DiDomenico, G. E., Brogan, L., Fein, J. A., Warrier, V., Guloksuz, S., & Barzilay, R. (2024). Classification of suicide attempt risk using environmental and lifestyle factors in 3 large youth cohorts. *JAMA Psychiatry*, *81*(10), 1020–1029. 10.1001/jamapsychiatry.2024.1887 39018056 10.1001/jamapsychiatry.2024.1887 PMC11255979

[CR56] Walsh, C. J., Rosenberg, S. L., & Hale, E. W. (2022). Obstetric complications in mothers with ADHD. *Front Reprod Health*, *4*, 1040824. 10.3389/frph.2022.1040824 36419963 10.3389/frph.2022.1040824 PMC9678343

[CR57] Weber, B. J., & Chapman, G. B. (2005). The combined effects of risk and time on choice: Does uncertainty eliminate the immediacy effect? Does delay eliminate the certainty effect? *Organizational Behavior and Human Decision Processes*, *96*(2), 104–118. 10.1016/j.obhdp.2005.01.001

[CR58] Wild, C. P. (2005). Complementing The genome with an exposome: The outstanding challenge of environmental exposure measurement in molecular epidemiology. *Cancer Epidemiology Biomarkers & Prevention*, *14*(8), 1847–1850. 10.1158/1055-9965.Epi-05-0456 10.1158/1055-9965.EPI-05-0456 16103423

