# Supplementary material for: Household Adversity, Day to Day Experiences, and Birth/Pregnancy Complications are Associated with Delay Discounting: Findings from the Adolescent Brain Cognitive Development Study
Source: Res Child Adolesc Psychopathol. 2026 Mar 2;54(2):42. doi: 10.1007/s10802-026-01437-y (PMC12953353; doi:10.1007/s10802-026-01437-y)
Supplement: Supplementary file 1 [file 10802_2026_1437_MOESM1_ESM.docx]

Supplemental Table 1. Associations of Exposome Factors with Delay Discounting Metrics Controlling for Number of Non-Systematic Reversals

|  | Log k | Log-Calculated AUC |  |
| --- | --- | --- | --- |
|  | β | se | p | FDR- adjusted p | Marginal R2(%) | β | se | p | FDR- adjusted p | Marginal R2 (%) |  |
| Model 1 |  |  |  |  |  |  |  |  |  |  |  |
| Household Adversity | 0.135 | 0.044 | 0.002 | 0.007 | 0.103 | -0.008 | 0.002 | 0.001 | 0.003 | 0.103 |  |
| Neighborhood Adversity | 0.034 | 0.052 | 0.514 | 0.617 | 0.011 | -0.002 | 0.003 | 0.497 | 0.617 | 0.011 |  |
| Positive Day-to-Day Experiences | -0.196 | 0.039 | 4.16E-07 | 2.50E-06 | 0.274 | 0.011 | 0.002 | 4.59E-08 | 5.51E-07 | 0.294 |  |
| State Conservatism/Ruralness | -0.022 | 0.050 | 0.663 | 0.723 | -0.004 | <0.001 | 0.003 | 0.983 | 0.983 | -0.009 |  |
| Family Values | -0.091 | 0.038 | 0.017 | 0.040 | 0.070 | 0.004 | 0.002 | 0.056 | 0.096 | 0.032 |  |
| Birth/Pregnancy Complications | 0.064 | 0.039 | 0.101 | 0.151 | 0.037 | -0.004 | 0.002 | 0.034 | 0.068 | 0.055 |  |
| Model 2 |  |  |  |  |  |  |  |  |  |  |  |
| Household Adversity | 0.052 | 0.048 | 0.279 | -- | 0.011 | -0.003 | 0.003 | 0.198 | -- | 0.013 |  |
| Neighborhood Adversity | 0.023 | 0.053 | 0.666 | -- | 0.005 | -0.001 | 0.003 | 0.639 | -- | 0.005 |  |
| Positive Day-to-Day Experiences | -0.178 | 0.040 | 1.18E-05 | -- | 0.202 | 0.010 | 0.002 | 2.53E-06 | -- | 0.214 |  |
| State Conservatism/Ruralness | -0.024 | 0.047 | 0.608 | -- | -0.004 | 0.001 | 0.003 | 0.827 | -- | -0.010 |  |
| Family Values | -0.074 | 0.039 | 0.055 | -- | 0.047 | 0.003 | 0.002 | 0.146 | -- | 0.028 |  |
| Birth/Pregnancy Complications | 0.066 | 0.040 | 0.097 | -- | 0.045 | -0.004 | 0.002 | 0.038 | -- | 0.061 |  |

Note. Bold values indicate statistical significance. Model 1 adjusted for child age, sex, parent-reported child race, household income, parent education, and number of non-systematic reversals, and included exposome factor in separate models. Model 2 adjusted for the same covariates and included all the exposome factors in the same model. FDR-adjusted p values are reported for Model 1.
